# COVID-19 Vaccine Boosters: The Good, the Bad, and the Ugly

**DOI:** 10.3390/vaccines9111299

**Published:** 2021-11-09

**Authors:** Piotr Rzymski, Carlos A. Camargo, Andrzej Fal, Robert Flisiak, Willis Gwenzi, Roya Kelishadi, Alexander Leemans, Juan J. Nieto, Ahmet Ozen, Matjaž Perc, Barbara Poniedziałek, Constantine Sedikides, Frank Sellke, Emilia C. Skirmuntt, Anzhela Stashchak, Nima Rezaei

**Affiliations:** 1 Department of Environmental Medicine, Poznan University of Medical Sciences, 60-806 Poznań, Poland; bpon@ump.edu.pl; 2 Universal Scientific Education and Research Network (USERN), https://usern.tums.ac.ir, Tehran 1417614411, Iran; ccamargo@partners.org (C.A.C.J.); amfal@wp.pl (A.F.); roya.kelishadi@gmail.com (R.K.); a.leemans@umcutrecht.nl (A.L.); juanjose.nieto.roig@usc.es (J.J.N.); ahmetozen_md@yahoo.com (A.O.); matjaz.perc@gmail.com (M.P.); c.sedikides@soton.ac.uk (C.S.); fsellke@lifespan.org (F.S.); khnmu_stashchak@ukr.net (A.S.); 3 Department of Emergency Medicine, Massachusetts General Hospital, Harvard Medical School, Boston, MA 02114, USA; 4 Collegium Medicum, Warsaw Faculty of Medicine, Cardinal Stefan Wyszyński University, 01-938 Warsaw, Poland; 5 Department of Infectious Diseases and Hepatology, Medical University of Bialystok, 15-540 Białystok, Poland; robert.flisiak1@gmail.com; 6 Biosystems and Environmental Engineering Research Group, Department of Agricultural and Biosystems Engineering, University of Zimbabwe, Mount Pleasant, Harare P.O. Box MP167, Zimbabwe; wgwenzi@agric.uz.ac.zw; 7 Department of Pediatrics, Child Growth and Development Research Center, Research Institute for Primordial Prevention of Non-Communicable Disease, Isfahan University of Medical Sciences, Isfahan 8174673441, Iran; 8 PROVIDI Lab, Image Sciences Institute, University Medical Center Utrecht, 3584 CX Utrecht, The Netherlands; 9 Instituto de Matemáticas, Universidade de Santiago de Compostela, 15782 Santiago de Compostela, Spain; 10 Department of Pediatric Allergy and Immunology, Marmara University School of Medicine, 34854 Istanbul, Turkey; 11 Faculty of Natural Sciences and Mathematics, University of Maribor, 2000 Maribor, Slovenia; 12 Department of Medical Research, China Medical University Hospital, China Medical University, Taichung 404332, Taiwan; 13 Center for Research on Self and Identity, School of Psychology, University of Southampton, Southampton SO17 1BJ, UK; 14 Alpert Medical School of Brown University, Division of Cardiothoracic Surgery, Rhode Island Hospital, Providence, RI 02905, USA; 15 Peter Medawar Building for Pathogen Research, Department of Zoology, University of Oxford, Oxford OX1 3SY, UK; emilia.skirmuntt@zoo.ox.ac.uk; 16 International Relations Office, Kharkiv National Medical University, 61000 Kharkiv, Ukraine; 17 Research Center for Immunodeficiencies, Children’s Medical Center, Tehran University of Medical Sciences, Tehran 1417614411, Iran; 18 Department of Immunology, School of Medicine, Tehran University of Medical Sciences, Tehran 1417614411, Iran

**Keywords:** immunology, pandemic, massive vaccinations, vaccine inequity, SARS-CoV-2

## Abstract

Pursuing vaccinations against COVID-19 brings hope to limit the spread of SARS-CoV-2 and remains the most rational decision under pandemic conditions. However, it does not come without challenges, including temporary shortages in vaccine doses, significant vaccine inequity, and questions regarding the durability of vaccine-induced immunity that remain unanswered. Moreover, SARS-CoV-2 has undergone evolution with the emergence of its novel variants, characterized by enhanced transmissibility and ability to at least partially evade neutralizing antibodies. At the same time, serum antibody levels start to wane within a few months after vaccination, ultimately increasing the risk of breakthrough infections. This article discusses whether the administration of booster doses of COVID-19 vaccines is urgently needed to control the pandemic. We conclude that, at present, optimizing the immunity level of wealthy populations cannot come at the expense of low-income regions that suffer from vaccine unavailability. Although the efficiency of vaccination in protecting from infection may decrease over time, current data show that efficacy against severe disease, hospitalization, and death remains at a high level. If vaccine coverage continues at extremely low levels in various regions, including African countries, SARS-CoV-2 may sooner or later evolve into variants better adapted to evade natural and vaccine-induced immunity, ultimately bringing a global threat that, of course, includes wealthy populations. We offer key recommendations to increase vaccination rates in low-income countries. The pandemic is, by definition, a major epidemiological event and requires looking beyond one’s immediate self-interest; otherwise, efforts to contain it will be futile.

## 1. Introduction

The COVID-19 vaccines were developed and authorized at an unprecedented pace. The first COVID-19 cases were reported in China in late 2019, whereas their etiological factor was identified in mid-January 2020 when its first whole-genome sequence was made publicly available by Chinese scientists. This development rapidly led to essential research on diagnostic methods, followed by studies of seroconversion, viral pathogenicity, and potential therapeutic targets [1,2,3]. At the same time, great effort has been expended in developing vaccine candidates based on attenuated or inactivated SARS-CoV-2 and those employing virus-like particles, mRNA, DNA, or recombinant proteins [4]. The first candidate to enter a phase I clinical trial was mRNA-1273 (Moderna Therapeutics, USA), with the first doses administered in mid-March 2020. At the turn of 2020–2021, two mRNA vaccines, BNT162b2 (BioNTech/Pfizer, Germany/USA) and mRNA-1273, became the first to be approved in the USA and European Union. This achievement would not have been made possible without use of innovative technologies, significant financial support, multiple trials running in parallel, and regulatory institutions working around the clock [5].

By early October 2021, twenty-two vaccines were approved in different parts of the world, including four adenoviral vector vaccines, two mRNA vaccines, ten vaccines based on inactivated SARS-CoV-2, five subunit vaccines, and one DNA vaccine. By October 2021, 45% of the world population had received at least one dose and one-third was fully vaccinated. In the European Union, four vaccines were available: BNT162b2 (delivered in two doses 21 days apart), mRNA-1273 (delivered in two doses 28 days apart), and two adenoviral vector vaccines, AZD-1222 (manufactured by Oxford/AstraZeneca and delivered in two doses 4–12 weeks) and AD26.COV2 (manufactured by Janssen/Johnson & Johnson and delivered in a single dose) [6,7,8,9].

Although one-third of the European Union population was still unvaccinated at the beginning of October 2021, the discussion of administering an additional COVID-19 vaccine doses (booster) was already in full swing. This discussion initially concerned immunocompromised patients whose response to vaccination is frequently lower, while some may not respond to initial vaccination at all and remain at high risk of severe COVID-19. As shown in Israel and USA, immunocompromised individuals may account for as much as 40–45% of hospitalized breakthrough cases [10,11]. Additionally, as illustrated in randomized clinical trials, administering a third mRNA vaccine to solid transplant recipients increases previously low antibody levels, improves cellular immunity, and decreases the share of individuals falling into the non-responders category [12,13,14]. It is now apparent that in immunocompromised patients, a three-dosing vaccination strategy is required for optimal immune generation. Therefore, recommendations to offer the additional COVID-19 vaccine dose to patients with primary or secondary immune deficiencies were generally accepted, justified, and issued in various regions in late summer 2021.

The recommendation to offer the additional vaccine dose for certain immunocompromised individuals cannot be extrapolated to the general population, which is expected to yield sufficient immune response following the original vaccination regimen. However, since late August 2021, Israel, in a pioneering move, was already recommending a third dose of BNT162b2 vaccine to all individuals aged 12 years and older [15]. Calls to offer booster doses to the general public have also become frequent in the European countries and the USA [16]. The rationale underlying these calls have been two-fold: (i) observations that serum antibody levels decrease within a few months following the completion of the initial vaccination regime, thus lowering protection against the infection [17,18,19]; and (ii) the emergence of more transmissible SARS-CoV-2 variants such as B.1.617.2 (delta variant), which increases the risk of breakthrough infection and could induce a higher viral load even in vaccinated individuals, at least within the first days after contracting the virus [20,21,22,23,24]. Although the European Medicine Agency stated that “there is no urgent need for the administration of booster doses of vaccines to fully vaccinated individuals” [25] in September 2021, a month later, it concluded that in the case of BNT162b2, “booster doses may be considered at least 6 months after the second dose for people aged 18 years and older” [26]; the agency expressed the same opinion on mRNA-1273 on 25 October 2021 [27]. These recommendations may have been met with relief by some national authorities in light of the autumn 2021 surge of infections in European countries, with the majority of cases caused by B.1.617.2. This SARS-CoV-2 variant, classified as a variant of concern (VOC), had become dominant in the USA and European Region by June/July 2021 [28]. By October 2021, the European Centre for Disease Prevention and Control distinguished two other VOCs: B.1.351 (beta) and P.1. (gamma), both showing community transmission. It also distinguished two variants of interest (VOI): B.1.621 (mu) and C.37 (lambda), both detected sporadically. The B.1.1.7 (alpha), dominant at the beginning of 2021, was considered to be a deescalated variant in Europe [29].

Although offering the booster to the general population will at some point become inevitable, we do not believe that these two reasons listed above suffice to justify such a recommendation at this moment. Here, we present arguments in favor and against the administration of a booster. We conclude that, although a booster strategy may be beneficial for wealthy nations in the short term, it is not only morally questionable but may also engender additional challenges for pandemic control if pursued at the expense of vaccinations in low-income countries. We strongly advocate prioritizing vaccination in poorly vaccinated regions of the world, as they constitute the hot spots for the SARS-CoV-2 evolution. Unvaccinated individuals remain the main drivers of viral spread in the population.

## 2. The Vaccine Booster Will Improve Protection against Infection

For individuals with no history of SARS-CoV-2 infection, the administration of the booster will represent a third (in the case of those vaccinated with AZD-1222, BNT162b2, or mRNA-1273) or second (in the case of those who previously received Ad26.COV2) exposure to the antigen. However, the boosters will also be offered to those who already had an infection. The short-term results of pre-authorization clinical trials have established high vaccine efficacy against infection under initial regimes. Observations of up to six months of individuals vaccinated with BNT162b2 showed a decline in such efficacy, estimated at a rate of approximately 6% every two months, with 84% efficacy four-to-six months after the second dose. However, these data could not predict the current situation, because the cut-off date for the analysis was 13 March 2021 [30], before the B.1.617.2 variant became dominant in various parts of the world. The results of clinical trials of mRNA-1273 have indicated that the efficacy against symptomatic infection 4–5 months after the second dose is 92%. However, again, the cut-off date of this trial (26 March 2021) preceded the global domination of the B.1.617.2 variant [30,31]. Nevertheless, there is clinical and post-authorization evidence that protection against infection gradually declines as serum antibody level decreases, whereas the circulation of highly transmissible variants such as B.1.617.2 may further increase the risk of contracting SARS-CoV-2 by vaccinated individuals [17,30,32,33,34].

As already shown in non-immunocompromised individuals, the administration of the booster effectively recalls specific immune responses to SARS-CoV-2 and increases serum antibody levels. This response likely restores the initial and considerable levels of protection against infection, even for the more highly transmissible variants, with potency to induce great viral loads in the upper respiratory tract [17,35,36,37,38]. Short-term evidence from Israel has demonstrated that rates of confirmed infections are lower in the booster group than in the vaccinated non-booster group [39]. Currently, there are no published data on clinical trials evaluating the efficacy of booster doses. Given that developed countries are facing a growing challenge of convincing unvaccinated individuals to receive a COVID-19 vaccine, offering boosters to vaccinated people represents a strategy for a more effective and local control of the pandemic. This strategy may be financially advantageous, particularly considering that, in the temperate zone, SARS-CoV-2 has revealed a strong seasonal pattern, with infections rising in the autumn–winter season [40]. Offering the booster may also contribute to keeping schools open, which is a boon for children’s education, as well as their mental and physical well-being [41,42]. Vaccine boosters offered to healthcare personnel, who were the first group to receive initial COVID-19 vaccine doses, will decrease the risk of SARS-CoV-2 transmission to patients, including vulnerable groups, and mitigate the disruption of the general healthcare system caused by the quarantine of SARS-CoV-2-positive healthcare workers. Moreover, the administration of the booster will likely improve the quality of life of COVID-19 high-risk groups who otherwise may experience higher stress levels during the autumn–winter waves of COVID-19 cases, hospitalizations, and deaths, all of which are further exaggerated by extensive media coverage and hype surrounding the danger of more transmissible SARS-CoV-2 variants.

## 3. Protection against Severe Disease after the Initial Vaccination Regime Remain High

Although serum antibody levels usually decrease within a few months after initial vaccination against COVID-19, this pattern cannot be directly translated into a gradual loss of immune protection. Firstly, no minimal threshold levels that confer protection against infection have been established so far. Secondly, the total anti-S or anti-S1-RBD IgG antibody levels that are most frequently quantified to assess the response to vaccination are not necessarily directly correlated with neutralization potencies. Thirdly, a vital role in antiviral response is played by the vaccine-induced adaptive cellular immunity [43]. Although the risk of breakthrough infections may increase over time in vaccinated individuals due to the waning of serum antibody levels and can be further elevated due to circulation of some more transmissible SARS-CoV-2 variants (e.g., B.1.617.2), this does not equal an increased risk of breakthrough diseases, especially ones leading to hospitalization or risk of death. Data originating from different parts of the world consistently show that COVID-19 vaccines retain high levels of protection against severe infections and death; that is, the risk of hospitalization and death due to severe COVID-19 is significantly higher in unvaccinated individuals, although its exact values may vary across populations due to different settings and types of administered vaccines [44,45,46,47,48,49,50,51,52]. For example, data collected by the US Centers for Disease Control and Prevention indicate that, in August 2021, unvaccinated individuals had a 6-fold higher risk of SARS-CoV-2 infection and an 11-fold higher risk of dying from COVID-19 [53]. Furthermore, the clinical trial on BNT162b2 that demonstrated a decline in efficacy against infection within six months after administering the second dose still reported an excellent overall efficacy (97%) against severe COVID-19. This pattern was also confirmed in a large retrospective study of nearly 3.5 million individuals conducted in the USA, which revealed a decline in the effectiveness of BNT162b2 vaccination against infection with the B.1.617.2 variant (from 93% during the first month after full vaccination to 53% after four months) but a stable effectiveness of 93% against hospitalization due to B.1.617.2 infection up to six months (maximum time of observation) following full vaccination [54]. These findings clearly indicate that, although the risk of infection may increase (likely due to a decline in serum antibody levels), the primary goal of vaccination (i.e., high level of protection from severe COVID-19 and death) is still met. Moreover, a clinical trial of Ad26.CoV2 vaccine demonstrated that a single dose elicits neutralizing antibodies that remain at a fairly stable level for at least nine months (maximum reported study time point) [37].

Even though studies have illustrated that initial viral loads of B.1.617.2 are comparable in vaccinated and unvaccinated individuals [21,55,56], these loads significantly decrease after 5–6 days from infection in the former group [21]. This decrease indicates that vaccine-induced cellular immunity offers protection from progression to more severe COVID-19. Ultimately, vaccine efficacy against infection may decrease over time, but protection against hospitalization and death remains high enough to prevent healthcare systems from being overwhelmed. In addition, a recent large study of contacts of persons infected with SARS-CoV-2 illustrated that vaccination is significantly reducing viral transmission, including that of B.1.617.2 variant, especially within the first three months from the second dose [57].

We should heed these facts. Vaccinology’s priority has always been to decrease the clinical severity of infection. The prevention of infection (whether symptomatic or asymptomatic) has been a secondary goal, while the complete pathogen eradication (highly improbable in the case of SARS-CoV-2) is the most challenging task [58]. Thus, mitigating the hospitalization rates and decreasing the overwhelming of healthcare systems must be prioritized. Unvaccinated individuals remain at the highest risk of severe COVID-19. We do not believe that optimizing the immunity of already vaccinated individuals should be a primary strategy to ease pressure on local hospitals.

At present, the durability of immune protection following vaccination remains unclear. At the same time, there is little evidence supporting a significant loss of protection against severe COVID-19, hospitalization, and death within the vaccinated population, at least within eight months since the completion of the initial vaccine regime. In other words, a primary goal of vaccination has been met and should now be urgently pursued in low-income countries.

## 4. Vaccine Inequity Will Place Large Numbers at High Risk of Severe COVID-19 and Death

The development and authorization of COVID-19 vaccines are intended to put the spread of SARS-CoV-2 under better control [5]. However, the efficient distribution of vaccine doses in different parts of the world represents a major challenge. The pandemic, by definition, is a major epidemiological event occurring over a wide geographic area and should be treated as such when pursuing global COVID-19 vaccination efforts. So far, this has clearly not been the case. While developed countries have already considered the recommendation of COVID-19 vaccine booster doses, at the current rate of vaccination, low-income countries will not likely achieve substantial protection levels until at least 2023 [59]. The aim of initiatives such as COVID-19 Vaccines Global Access (COVAX) is equitable access to COVID-19 vaccines in low-to-middle-income countries. The initial goal of COVAX was to deliver 2 billion vaccine doses by the end of 2021. However, by October 2021, only 311 million (15.5% of the target) had been shipped [60]. The direct response from wealthy nations has also been subdued. For example, though the USA has pledged to donate at least 1.1 billion doses of COVID-19 vaccine doses for global use before 2023, only 200 million had been shipped by late October 2021 [61].

Although wealthy nations have publicly declared their support, the disparity in vaccination rates in different parts is substantial. The World Health Organization has repeatedly urged political leaders to hold off on the administration of boosters in order to enable every nation to vaccinate at least 40% of the population. This call will likely be (or already has been) discarded by several developed countries, some of which have destroyed an excess of unused doses, sold them to other wealthy nations, or imposed vaccine export bans. Commentators have called these actions a “moral outrage” and a “crime against humanity” [62].

The prevalence of fully vaccinated Africans by October 2021 amounted to only 4%, 16-fold and 12-fold lower compared to the vaccination rate in the European Union and North America, respectively (Table 1). This has rendered a significant share of individuals in low-income regions fully vulnerable to SARS-CoV-2 infection and its consequences. Such regions lack the capacity to independently cope with epidemiological outbreaks due to high poverty rates, anemic healthcare and social security systems, shortages in diagnostic equipment and medicines, weak research capacities, and low epidemiological surveillance [63].

The reported number of COVID-19 cases and deaths does not fully reflect the pandemic’s impact in the African region due to substantially smaller testing capacities on this continent compared to, for example, Europe, Oceania, and North America [65,66]. An efficient COVID-19 vaccination campaign can avert numerous hospitalizations and deaths. In the United States alone, vaccination was estimated to prevent 1.25 million hospitalizations and 279 thousand COVID-19-related deaths by the end of June 2021 [67]. An efficient vaccination campaign would save even more lives in Africa, as its population is over 3.5-fold larger than that of USA. Moreover, COVID-19 has far-reaching impacts on various aspects of society including socio-economic development. The limited access to vaccination may not only increase the morbidity and mortality associated with COVID-19 but also adversely affect the already fragile economies of low-income countries, making post-COVID-19 recovery even more difficult.

## 5. The Vaccine Inequity Will Drive the SARS-CoV-2 Evolution

There is evidence that focusing vaccination efforts on unvaccinated individuals is a strategy to suppress the mutation dynamics of SARS-CoV-2 [68]. As shown in countries dominated by the B.1.617.2 variant, there is a significant negative correlation between fully vaccinated rate and mutation frequency (*Mf*), with the highest values of *Mf* observed for populations with vaccination rates below 10% [68]. These findings should represent a wake-up call for wealthy nations—and all political and public health leaders—to pursue humanitarian aid in the distribution of the COVID-19 vaccine. An alternative approach to suppressing the *Mf* includes implementing strict non-pharmaceutical mitigation strategies based on use of personal protection equipment, hand washing, and social distancing. However, the implementation of such control measures has been met with significant challenges and resistance in developed countries; in regions such as Africa (featuring the lowest vaccination rate in the world), these challenges are even more severe due to poverty and strong socio-cultural norms and practices, including religious practices, gender disparities, and beliefs [63]. Additionally, since the first COVID-19 vaccines were authorized, SARS-CoV-2 has undergone adaptation, increasing its transmissibility and potency to induce high viral loads. According to some reports, the basic reproduction number for the B.1.617.2 variant is up to 8, compared to 4.0 for B.1.1.7 (alpha variant) and <3.0 for variants dominant in 2020 [69,70]. Although B.1.617.2 can initially induce similar viral loads in vaccinated and unvaccinated individuals [19,53,54], the former group suppresses the SARS-CoV-2 replication significantly sooner, thus reducing mutation rates, limiting within-host adaptations, and curtailing further SARS-CoV-2 spread in the population [21,71]. Therefore, the reports on identified VOCs and VOIs should motivate continued primary vaccination in developed regions and a greater effort to reduce vaccine inequities in low-income countries as soon as possible. The high proportion of the unvaccinated population creates an environment that is more favorable to the infection, replication, and evolution of SARS-CoV-2, along with the concurrent threat of emergence of variants that possess multiple immune escape mutations. In addition, the emergence of variants such as B.1.351 (beta variant), B.1.525 (eta variant), B.1.621 (mu variant), P.2. (zeta variant), and P.3. (theta variant) characterized by the E484K mutation in the receptor-binding domain of the spike protein (which partially evades antibody neutralization in convalescent and vaccinated individuals) occurred prior to the implementation of massive COVID-19 vaccination programs [29]. Leaving the poorer parts of the world without COVID-19 vaccines may eventually present a threat for wealthy nations through the spontaneous emergence and global spread of variants that possess novel escape mutations, thus significantly decreasing vaccine efficacy against infection, severe COVID-19, and death.

## 6. Key Recommendations

In light of the fact that COVID-19 is a major epidemiological event and to promote equity in COVID-19 vaccine, we make the following recommendations:

1. Access to the vaccines developed to fight pandemics, including COVID-19, needs to be declared a human right.
2. The promotion of vaccine funding in low-income countries by global health agencies should be continued and strengthened.
3. Global health agencies and funding partners should organize global crowdfunding to support vaccine deliveries to low-income countries.
4. As part of their corporate social responsibility and contribution to the global fight against the pandemic, large pharmaceutical companies involved in the production of COVID-19 vaccines should consider a two-tier pricing system with a cross-subsidy. In this case, developed countries targeting booster vaccination will acquire vaccines at a higher price than their low-income counterparts, resulting in the former subsidizing the latter. This pricing model is conceptually similar to the block tariff system commonly used for water pricing and is anchored by the notion that access to COVID-19 vaccines is a human right.
5. Every individual receiving a booster dose in a developed country should be given a voluntary opportunity to financially support vaccination in low-income countries. Concurrently, public campaigns should be launched in developed countries to raise awareness of the global importance of COVID-19 vaccinations in low-income countries.
6. Export bans on COVID-19 vaccines should be lifted in developed countries, particularly for vaccines targeted at low-income countries.
7. A ban on trading COVID-19 vaccines between developed countries should be imposed. The excess doses should be donated to the COVID-19 vaccine humanitarian aid program.
8. Low-income countries should prioritize allocating financial resources and co-funding COVID-19 vaccination programs in collaboration with external partners. Co-funding ensures the local ownership of such COVID-19 vaccination programs and will avert the perpetuation of the donor syndrome prevalent in low-income regions such as Africa. However, to achieve this, financial governance and accounting systems in low-income countries will need to be strengthened to ensure transparency and the efficient use of local and donor funds.
9. Massive campaigns to fight vaccine hesitancy, educate on COVID-19 and its threats, promote vaccine uptake, and make better use of available vaccine doses need to be launched in low-income countries with support from the international scientific community, bringing together experience from vaccinations in developed countries and local specificity.
10. The high-quality production of COVID-19 vaccines in low-income countries should be supported to decrease the dependency of these countries on imports and donations. This capacity to produce high-quality vaccines should be extended to other human infections accounting for high human morbidity and mortality in low-income countries.

## 7. Conclusions

The administration of COVID-19 vaccine booster doses will restore high antibody levels and provide additional protection against SARS-CoV-2 infection. However, the initial vaccination regimes offer sufficiently high protection from severe disease, hospitalization, and death despite the emergence of highly transmissible SARS-CoV-2 variants such as B.1.617.2. At the same time, there are billions of unvaccinated individuals worldwide, the majority of whom reside in low-income regions and remain the main drivers of the pandemic. We should prioritize vaccinating the unvaccinated individuals and decreasing vaccine inequities rather than optimizing immunity levels in wealthy nations. If vaccine coverage continues to remain inadequate in low-income countries, SARS-CoV-2 may sooner or later evolve into variants better adapted to evade natural and vaccine-induced immunity, ultimately endangering wealthy populations. Containing the pandemic requires looking beyond one’s immediate self-interest.

## Figures and Tables

**Table 1:** The proportion of fully and partially vaccinated and unvaccinated individuals in different world by 1 October 2021. Based on [64].

Region	Fully Vaccinated	Partially Vaccinated	Unvaccinated
	[%]		
Africa	4	2	94
Asia	37	15	48
Oceania/Australia	32/43	16/20	52/37
Europe/European Union	52/63	4/4	44/33
North America	47	10	43
South America	41	20	39
World	34	12	54
